# A case of a gastric granular cell tumor preoperatively diagnosed and successfully treated by single-incision laparoscopic surgery

**DOI:** 10.1186/s40792-020-00809-w

**Published:** 2020-02-27

**Authors:** Atsushi Yasuda, Takushi Yasuda, Haruhiko Imamoto, Yoko Hiraki, Kohta Momose, Hiroaki Kato, Mitsuru Iwama, Osamu Shiraishi, Masayuki Shinkai, Motohiro Imano, Yutaka Kimura

**Affiliations:** 1 Department of Surgery, Kindai University Faculty of Medicine, 377-2 Ohno-higashi, Osaka-Sayama, Osaka, 589-8511 Japan; 2 Cancer Center, Kindai University Hospital, 377-2 Ohno-higashi, Osaka-Sayama, Osaka, 589-8511 Japan

**Keywords:** Granular cell tumor, Stomach, SILS, EUS-FNAB

## Abstract

**Background:**

Granular cell tumors (GCT) in the gastrointestinal tract are rare. Herein, we describe a case of a gastric GCT diagnosed preoperatively by endoscopic ultrasound-guided fine needle aspiration biopsy (EUS-FNAB) and successfully resected by single-incision laparoscopic surgery (SILS).

**Case presentation:**

A 46-year-old Japanese woman had a tumor located in the angle of the stomach that was approximately 1.5 cm in diameter. Abdominal computed tomography (CT) revealed a submucosal tumor (SMT), which was finally diagnosed as a gastric GCT using EUS-FNAB. The tumor was not identified by CT 1 year and 4 months before diagnosis; therefore, because there was a possibility that the tumor was malignant, we performed surgical wedge resection using SILS. The patient had an uneventful recovery postoperatively and was discharged without complications 3 days after surgery. The tumor was pathologically diagnosed as a benign GCT that remained within the muscular layer. No recurrence or complications have occurred in the first 16 months since the surgery.

**Conclusion:**

Because gastric GCTs are generally benign and are rarely associated with lymph node metastasis, SILS seems to be a safe and feasible surgical approach for treating GCTs.

## Background

Granular cell tumors (GCTs), which were first reported by Weber in 1854 [1], are infrequent mesenchymal soft-tissue tumors derived from Schwann cells and are generally benign [2–4]. They can be found at many sites, including the skin, tongue, subcutaneous tissues of the chest, upper extremities, and female genital area [5, 6]. However, GCTs in the gastrointestinal tract are uncommon and comprise approximately 4–6% of all GCTs [6]. The most common location for GCTs in the gastrointestinal tract is the esophagus, while the second most common site is the colon [7]; gastric localization is very uncommon for GCTs.

Herein, we describe a case of GCT that was successfully diagnosed by endoscopic ultrasound-guided fine needle aspiration biopsy (EUS-FNAB) preoperatively and resected by single-incision laparoscopic surgery (SILS), which is a minimally invasive surgical technique.

## Case presentation

**Fig. 1:**
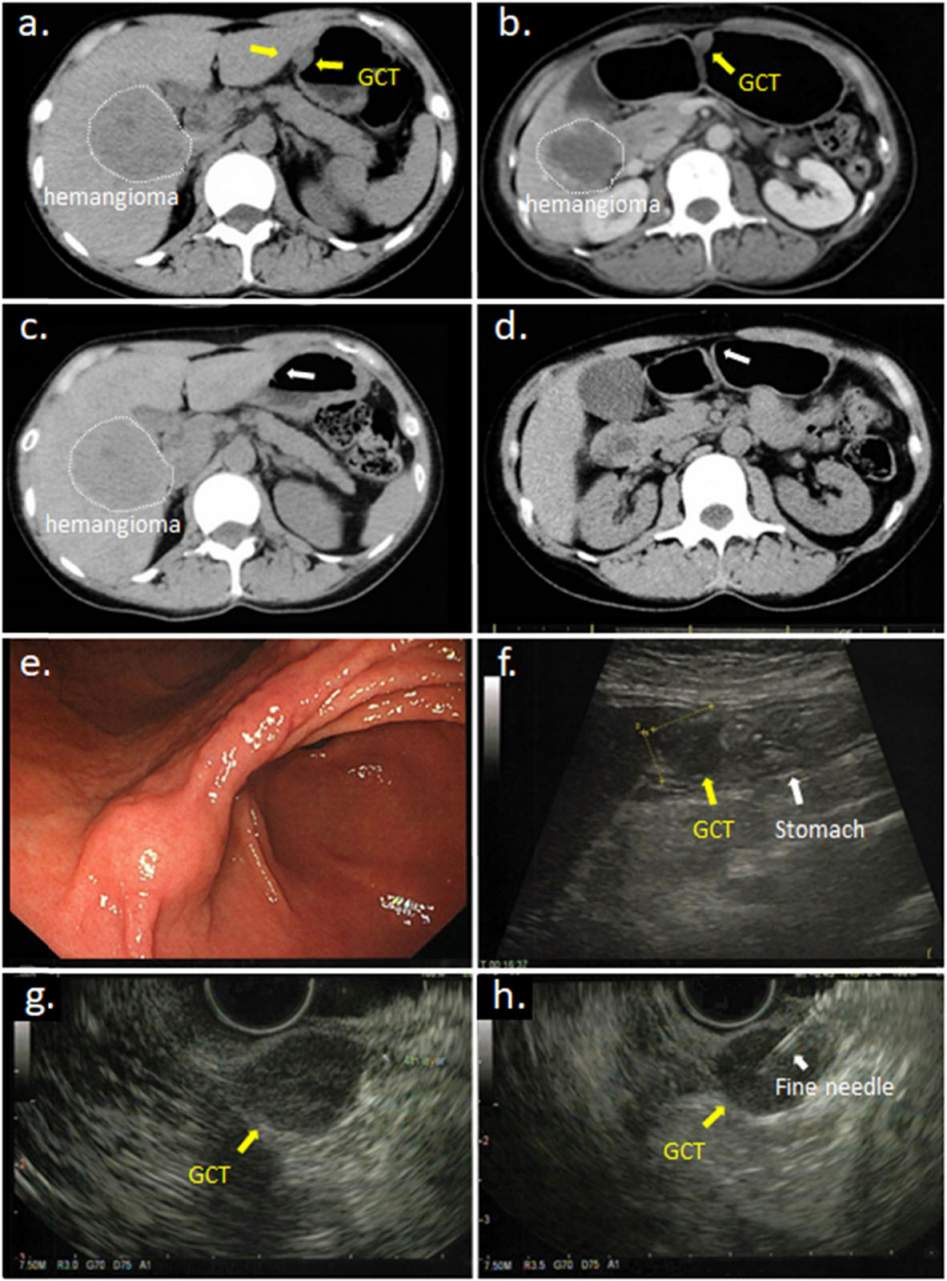
**a** Abdominal computed tomography (CT) revealed a solid extramural tumor situated at the lesser curvature of the lower body of the stomach (yellow arrow). The portion surrounded by white dotted line is hepatic hemangioma. **b** CT using foaming agent showed it more clearly. **c** The CT scan performed one year ago and **d** 4 years ago showed the tumor did not exist (white arrow). **e** Gastroscopy revealed submucosal tumor at the anterior side of the angle of the stomach. **f** Abdominal ultrasound sonography showed a low echoic mass measuring 15 mm in diameter existed in contact with the stomach. **g** Endoscopic ultrasound sonography (EUS) showed that the tumor of about 15 mm in diameter occurred from the fourth layer of the gastric wall and **h** the needle of EUS-guided fine needle aspiration biopsy (EUS-FNA) surely punctured to the tumor

**Fig. 2:**
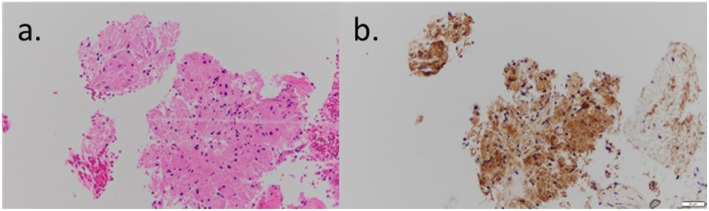
The specimens by endoscopic ultrasound sonography-guided fine needle aspiration biopsy (EUS-FNAB) using **a** hematoxylin-eosin staining (× 400) and **b** immunohistochemical staining for S-100 protein (× 400)

A 46-year-old Japanese woman underwent computed tomography (CT) or ultrasonography (US) examinations every year since the detection of a giant hepatic hemangioma (Fig. 1a, b). A follow-up CT scan incidentally detected a tumor that was approximately 1.5 cm in diameter at the angle of the stomach in the anterior gastric wall (Fig. 1a, b), which had not appeared in the CT image 1 year and 4 months previously (Fig. 1c, d). The subsequent examinations by upper gastrointestinal endoscopy (UGE) and abdominal US revealed that it was a submucosal tumor (SMT) with homogeneous low echoes (Fig. 1e, f). Physical examination, biochemical data, tumor markers (including carcinoembryonic antigen (CEA), carbohydrate antigen 19-9 (CA19-9), cancer antigen 125 (CA125), and squamous cell carcinoma (SCC)), and chest and abdominal X-ray films were all within normal limits. Endoscopic US (EUS) (20 MHz) was also performed to evaluate clinical characteristics of this tumor; it revealed that the tumor had developed from the fourth layer of the gastric wall, which corresponds to the muscular layer, and had homogeneous low echoes (Fig. 1g). A small amount of tissue sample was taken using the EUS-FNAB technique (Fig. 1h) and was provided for pathological examinations. As a result, the pathological findings showed several spindle-shaped cells with small circular nuclei and a granular eosinophilic cytoplasm. Furthermore, immunohistochemical staining revealed that S-100 was positive (Fig. 2a, b); c-kit, CD34, and desmin were negative. Based on these results, the tumor was diagnosed as a GCT.

Conservative management was considered as a therapeutic option due to the benign nature of most GCTs; in addition, the tumor size was smaller than 2 cm and no swollen lymph nodes were observed. However, it was clear that this tumor was new and had grown quickly between CT examinations; thus, there was a possibility that this tumor was malignant. Consequently, we decided to resect the tumor using local resection without lymph node dissection. Considering the cosmetic appearance of the wound in the young female patient, we decided to use the SILS method to perform a minimally invasive surgery.

### Surgical procedure by SILS

**Fig. 3:**
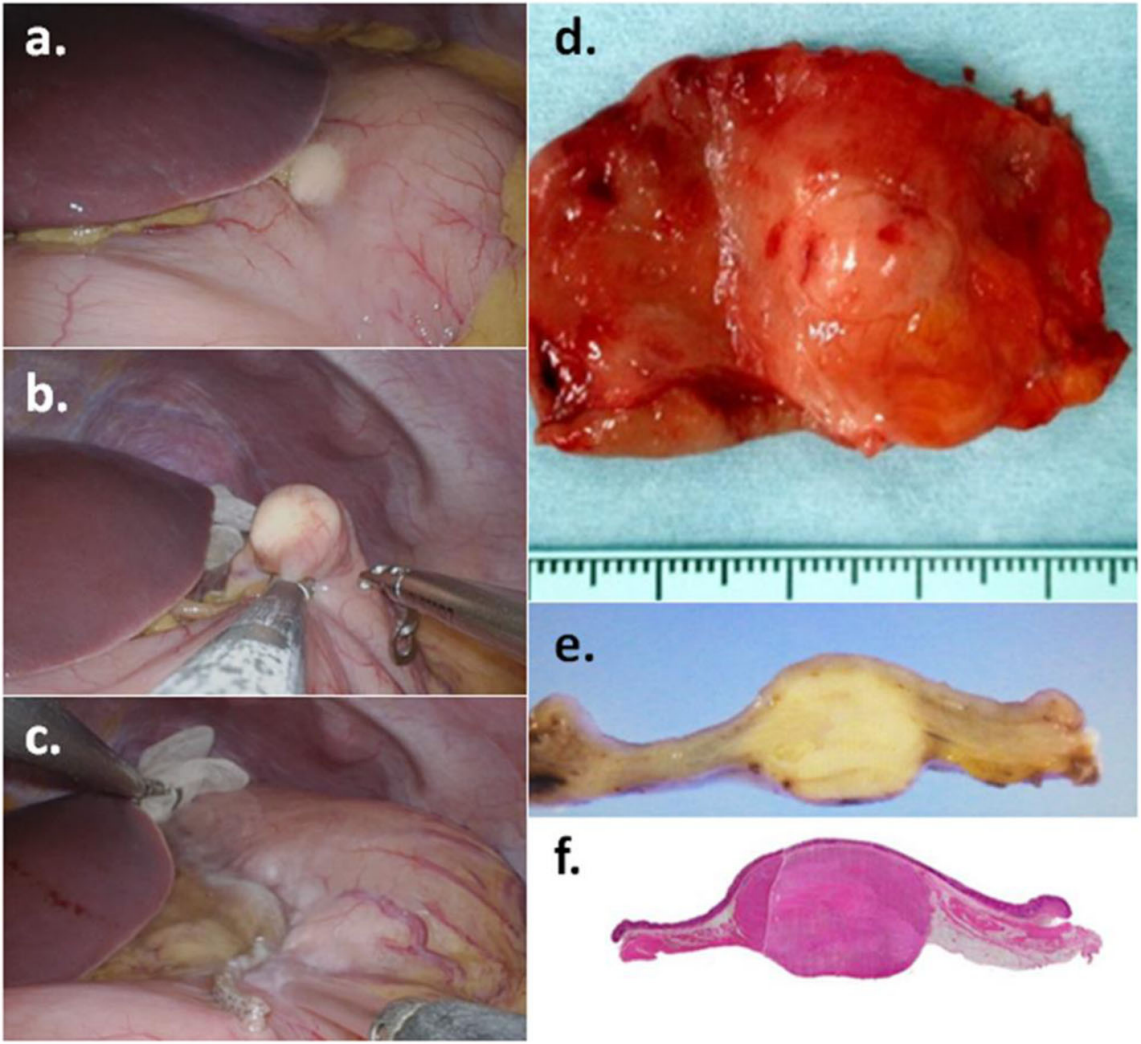
The tumor could be seen at the angle of the stomach (**a**) and was resected with auto suturing device; Signia™ Stapling System (**b**, **c**). The tumor was an elastic soft mass with a thin fibrous capsule and measured 15 × 15 mm in diameter (**d**, **e**, **f**)

We placed a 3-cm skin incision on the transumbilical plane and equipped the incision site with the Lap Protector mini™ (Hakko Co. Ltd., Tokyo, Japan) and EZ access (Hakko Co. Ltd., Tokyo, Japan). We used an 11-mm trocar and two 5-mm trocars and inserted them into the abdominal cavity through the EZ access. No other assist ports were placed. When the flexible scope for laparoscopic surgery was inserted into the abdominal cavity, the tumor could be seen at the angle of the anterior wall of the stomach as a small white colored nodule slightly protruding from the gastric wall. It was covered with a thin serosal capsule and had a smooth surface (Fig. 3a). The lesser omentum near the tumor was dissected using a bipolar-tissue sealing system (ENSEAL®G2 with Articulating head, Ethicon Endo-Surgery Inc., Cincinnati, OH) in order to perform complete resection with an adequate surgical margin. The cutting line was confirmed with forceps before resection of the tumor (Fig. 3b). After these procedures, the tumor was resected with an auto-suturing device (Signia™ Stapling System, Covidien Japan, Inc., Tokyo, Japan) (Fig. 3c). Finally, after verifying that hemostasis was achieved and that there was no suture leakage of the resected stump, the wound was closed in two layers, using a standard protocol.

**Fig. 4:**
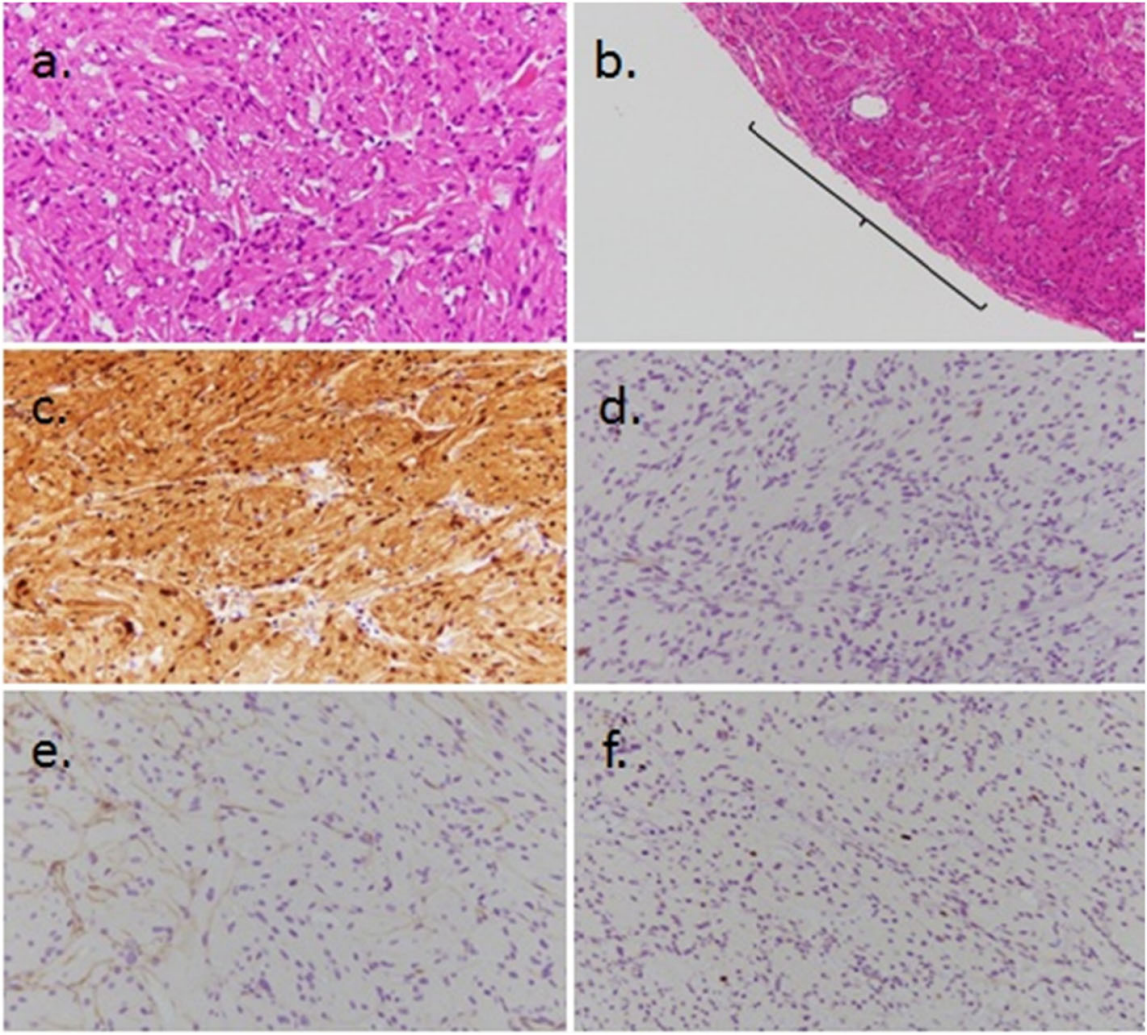
**a** The specimens of surgically resected tumor using hematoxylin-eosin staining (× 400). **b** The capsule of the tumor was partially lacking using hematoxylin-eosin staining (× 100). **c** The specimens of surgically resected tumor using immunohistochemical staining for S-100 protein (× 400). **d** The specimens using immunohistochemical staining for c-kit (× 400). **e** The specimens using immunohistochemical staining for CD34 (× 400). **f** The specimens using immunohistochemical staining for Ki-67 (× 400)

The resected tumor was an elastic soft mass with a thin fibrous capsule, was 15 × 15 mm in size (cross-section), and was located in the proper muscular layer of the stomach wall (Fig. 3d–f). Hematoxylin and eosin staining revealed circular-shaped or spindle-shaped cells with small circular nuclei and a granular eosinophilic cytoplasm, which was proliferated densely within the tumor (Fig. 4a); the tumor had only a partial capsule (Fig. 4b). Immunohistochemical staining showed that the S-100 protein was detected in nearly all cells of the tumor (Fig. 4c), while c-kit and CD34 were negative (Fig. 4d, e). In addition, Ki-67 expression was observed in less than 1% of cells (Fig. 4f). Based on the above results, the resected tumor was diagnosed as a benign GCT. The patient had an uneventful recovery postoperatively and was discharged without complications three days after surgery. No recurrence or complications have occurred in the last 16 months since the surgery.

## Discussion

We have reported a case of a GCT that was diagnosed preoperatively using EUS-FNAB and treated with local excision using the SILS technique.

In recent years, advancements in endoscopy-related equipment have led to frequent detection of small SMTs. However, in cases of small SMTs, it is difficult to determine the most effective treatment strategy, even when considering the treatment policies and clinical guidelines for gastric SMTs and GCTs [8]; that is, many issues still remain. First, it may be challenging to diagnose SMT qualitatively and accurately. According to the guidelines, we added EUS and EUS-FNAB due to the presence of an SMT less than 2 cm in diameter and confirmation of tumor enlargement, in comparison to previous images. Fortunately, we were able to diagnose a GCT based on the pathological examination of the EUS-FNAB sample; however, in some cases, it can be challenging to obtain sufficient tissue to make an accurate pathological diagnosis. In Japan, there are only 9 known cases that have used EUS-FNAB to diagnose GCT. Smaller tumors are more difficult to diagnose accurately. Furthermore, because GCTs of the stomach are rare, there are no distinctive EUS images that are used to characterize GCTs. Search for the term *gastric granular cell tumor* in PubMed and Ichushi-Web (Japan Medical Abstracts Society; JAMAS) from inception to 2019 yielded 39 cases, including ours, in Japan (Table 1) [9–12] and 32 cases in other countries [13, 14].

**Table 1:** Case reports of GCTs of the stomach in Japan

Case	Author	Year	Treatment	Size (mm)	Pathological depth	Malignancy	Concomitant lesion
1	Tuneyosi	1978	*	7	*		
2	Tuneyosi	1978	*	7	*		
3	Tuneyosi	1978	*	15	*		
4	Takagi	2004	*	2	*		
5	Takagi	2004	Endoscopic resection	6	*		
6	Tanimura	1980	Endoscopic resection	10	≦sm		
7	Asagi	1981	Endoscopic resection	12	≦sm		
8	Eda	1988	Endoscopic resection	17	≦sm		
9	Toyohara	1991	Endoscopic resection	13	≦sm		
10	Yasuda	1995	Endoscopic resection	8	≦sm		
11	Matsumoto	1996	Endoscopic resection	6	≦sm		
12	Wakasugi	1996	Endoscopic resection	6	≦sm		
13	Nakazawa	2000	Endoscopic resection	10	≦sm		
14	Katujima	2003	Endoscopic resection	12	≦sm		
15	Kato	2012	Endoscopic resection	20	≦sm		
16	Ozawa	2010	Endoscopic resection	9	mp		
17	Tomura	1969	Gastrectomy	10	≦sm		Neurinoma
18	Saito	1975	Gastrectomy	3	≦sm		Gastric cancer and leiomyoma
19	Arima	1978	Gastrectomy	10	≦sm		Gastric ulcer
20	Hamada	1984	Gastrectomy	13	≦sm		
21	Ozaki	1988	Gastrectomy	8	≦sm		
22	Tuchida	1989	Gastrectomy	20	≦sm		Gastric ulcer
23	Xuan	1992	Gastrectomy	12	≦sm		Gastric cancer
24	Hirai	1995	Gastrectomy	20	≦sm		
25	Fujii	2001	Gastrectomy	10	≦sm		Gastric cancer
26	Eguchi	2002	Gastrectomy	*	≦sm		Gastric cancer and malignant lymphoma
27	Nishimori	1991	Gastrectomy	18	mp		Duodenal ulcer
28	Matsumoto	1996	Gastrectomy	70	mp	(+)	
29	Maekawa	2003	Gastrectomy	15	mp		Esophageal GCT
30	Kobayasi	1971	Nucleation	10	≦sm		Gastric ulcer
31	Nagaoka	1997	Nucleation	20	≦sm	(+)	
32	Ikeda	1977	Wedge resection	20	≦sm		
33	Yamaguti	1989	Wedge resection	15	≦sm		Gastric cancers
34	Sano	2008	Wedge resection	11	≦sm		
35	Nomura	1973	Wedge resection	10	mp		Gastric ulcer
36	Sano	2008	Wedge resection	12	mp		
37	Niwatari	2009	Wedge resection	45	mp		
38	Iwanaga	2019	Wedge resection (LECS)	10	≦sm		
39	Our case	2019	Wedge resection	15	mp		

The second issue is that a routine treatment strategy has not been established for GCTs. It is important and valuable to get an accurate diagnosis, but it is not required when deciding the appropriate treatment plan. Although GCTs are benign in most cases, the possibility of malignancy of the SMT should not be denied preoperatively. Accordingly, GCTs should be resected and, ideally, detected early and excised using minimally invasive procedures.

The third issue involves the resection of GCTs; there is controversy regarding the resection method that should be used for GCTs. According to previous Japanese reports, endoscopic resection was performed in 12 of 34 resected gastric GCT cases, excluding our case, and no local recurrence was observed. Twenty-five of these 34 GCTs (75.6%) originated pathologically from the mucosal or submucosal layer of the stomach wall, and all tumors were ≤ 2 cm in diameter (Table 1) [9–12]; based on these findings, endoscopic resection might be a feasible treatment option. However, tumor invasion into the proper muscular layer was observed in 7 of 34 resected cases, excluding one with an unknown tumor depth; 5 of these tumors which were < 20 mm in size. Considering these data and that endoscopic resection was mainly performed for diagnostic purposes, endoscopic resection should be performed for GCTs with caution.

On the other hand, as shown in Table 1, 23 GCTs were resected surgically in Japan. Fourteen of these tumors were resected surgically due to their size or the presence of comorbidities. Gastrectomy was performed in 13 cases, wedge resection was performed in 8 cases, and enucleation was performed in 2 cases (Table 1) [9–12]. Although the short-term and long term outcomes were inconsistent in the previous reports, the cases did not show recurrence, regardless of the type of surgery performed; thus, we considered that if the tumor is completely removed, the minimum surgical margin seems to be adequate.

The fourth issue concerns the need for lymphadenectomy. There were some reports of malignant GCTs of the esophagus and the breast with regional lymph node metastasis; thus, regional lymph node dissection and tumor resection are recommended in cases of malignant GCTs [15, 16]. However, regarding GCTs in other regions, including the stomach, no studies have reported GCTs with lymph node metastasis. This is most likely because gastric GCTs are rarely malignant. There are only two cases of malignant gastric GCTs reported in Japan [14, 17], and lymph node metastasis was not observed in either of these cases. Due to the limited reported cases of malignant gastric GCTs, the clinical feasibility of lymph node dissection is not definitive.

The final issue regards the determination of the proper surgical approach; open surgery, conventional laparoscopic surgery, and SILS are the most commonly used surgical techniques for GCTs. Recently, some reports have resected gastric SMTs, including gastrointestinal stromal tumors, using the SILS technique [18–20]; we also used the SILS technique, which was established in 2006. As described above, local resection is acceptable for benign or low-grade malignant gastric SMTs without lymph node metastasis. In our case, the tumor was small (< 2 cm in diameter) and was located in the anterior wall of the angle of the stomach, which is easily accessible. Therefore, we decided that SILS could be used safely and reliably to resect the tumor. The SILS approach has great advantages, in terms of alleviation of surgical stress and better cosmetic outcomes; thus, it should be taken into consideration as a feasible surgical approach in cases of small tumors that are non-malignant (or have a very low risk of becoming malignant) or when lymph node dissection is not necessary. In the recent years, laparoscopy and endoscopy cooperative surgery (LECS) [21] was a good indication for this kind of SMT and could be one of the options in this case; however, in this study, we adopted the SILS method that has been used and become familiar.

## Conclusion

In conclusion, our case study suggests that the SILS technique is a safe and feasible surgical procedure for gastric GCTs. However, because the necessity of lymph node dissection is still controversial, the malignant potential of gastric GCTs remains unclear, and the reliable implementation of SILS requires surgeons to have adequate skills and experience for conventional laparoscopic surgery, indications for surgery should be carefully tailored for each case. Because gastric GCTs are rare, it is also important to accumulate cases of GCTs and continually examine optimal treatment methods, in the future.

## Data Availability

All datasets supporting the conclusions of this article are included within the article.

## Abbreviations

CT: Computed tomography (CT)
EUS-FNAB: Endoscopic ultrasound-guided fine needle aspiration biopsy
GCTs: Granular cell tumors
SILS: Single-incision laparoscopic surgery
SMT: Submucosal tumor
US: Ultrasonography (US)
